# Diversity, Biocontrol, and Plant Growth Promoting Abilities of Xylem Residing Bacteria from Solanaceous Crops

**DOI:** 10.1155/2014/296521

**Published:** 2014-05-19

**Authors:** Gauri A. Achari, Raman Ramesh

**Affiliations:** ^1^ICAR Research Complex for Goa, Old Goa, Goa 403 402, India; ^2^Department of Microbiology, Goa University, Taleigao Plateau, Goa 403206, India

## Abstract

Eggplant (*Solanum melongena* L.) is one of the solanaceous crops of economic and cultural importance and is widely cultivated in the state of Goa, India. Eggplant cultivation is severely affected by bacterial wilt caused by *Ralstonia solanacearum* that colonizes the xylem tissue. In this study, 167 bacteria were isolated from the xylem of healthy eggplant, chilli, and *Solanum torvum* Sw. by vacuum infiltration and maceration. Amplified rDNA restriction analysis (ARDRA) grouped these xylem residing bacteria (XRB) into 38 haplotypes. Twenty-eight strains inhibited growth of *R. solanacearum* and produced volatile and diffusible antagonistic compounds and plant growth promoting substances *in vitro*. Antagonistic strains XB86, XB169, XB177, and XB200 recorded a biocontrol efficacy greater than 85% against BW and exhibited 12%–22 % increase in shoot length in eggplant in the greenhouse screening. 16S rRNA based identification revealed the presence of 23 different bacterial genera. XRB with high biocontrol and plant growth promoting activities were identified as strains of *Staphylococcus* sp., *Bacillus* sp., *Streptomyces* sp., *Enterobacter* sp., and *Agrobacterium* sp. This study is the first report on identity of bacteria from the xylem of solanaceous crops having traits useful in cultivation of eggplant.

## 1. Introduction

*Ralstonia solanacearum* is a vascular wilt pathogen that belongs to the *β* subdivision of the Proteobacteria [1] and is one of the most destructive plant pathogens causing bacterial wilt (BW) in many crop plants. It has broad host range and infects around 54 plant families and 450 plant species [2]. This pathogen also has a wide geographical distribution ranging from tropical, subtropical, and warm temperate regions of the world [3]. Cultivation of eggplant in the coastal state of Goa, India, is severely affected by BW leading to 30–100% crop loss [4]. The bacterium infects the plant through root cracks at the site of root emergence. Subsequently, the intercellular spaces of the root cortex and vascular parenchyma are colonized. Cell wall degrading exoenzymes disrupt the cell walls and facilitate its entry in the vascular system [5]. Inside the xylem vessels, the bacterial populations rapidly reach very high levels of 10^10^ cells/cm of stem [6]. High cell density and production of high molecular weight exopolysaccharides by* R. solanacearum* lead to clogging of xylem vessels, wilting, and eventually death of plant.

Xylem of healthy plants has been reported to be colonized by endophytic xylem residing bacteria (XRB) at low population levels and has been isolated from xylem of various crops, namely, citrus [7], sugar beets [8], maize [9], alfalfa [10], grape [11, 12], and Bermuda grass [13]. Several endophytic bacteria have been reported to originate from the rhizosphere soil, initially entering the host plant during germination and radicle development, through wounds or by colonizing the cracks formed in lateral root junctions when the endodermis and casparian strips are disrupted thus gaining an easy access to the stele [14, 15]. After their initial entry, depending on the endophytic colonization ability, bacteria may remain localized in the roots [16] or colonize intercellular spaces and vascular system [11] and move to the stems [17]. Few endophytes have been reported to be able to migrate to aerial plant parts through the vascular system passively with the transpirational flow or through additional assistance by production of cell wall degrading enzymes [18, 19]. These systemically migrated endophytes have been isolated from leaves [20], inflorescence [21], fruits [22], and seeds [23].

Among the several methods of plant disease management, biocontrol plays an important role particularly in the control of soil borne diseases. Biocontrol agents may be used as an alternative pathogen management strategy or can be combined with other management practices. Biological control not only helps in suppressing the disease and increasing crop yield but also has importance in reducing the environmental pollution due to use of chemical pesticides [24]. Several studies have shown that endophytic bacteria can be used as biocontrol agents against plant pathogens. The capability of colonizing internal host tissues and ability to produce volatile and diffusible substances which inhibit pathogen, induction of systemic resistance in the plant, and directly or indirectly promoting plant growth have made endophytes a valuable tool in agriculture to improve crop performance [18]. Endophytic biocontrol agents isolated from potato [25], tomato, chilli [26] and eggplant [27] have been used for management of BW. However, the wilt prevention ability of xylem residing bacteria of solanaceous crops that share an ecological niche with the BW pathogen has remained unexplored. This study was undertaken to identify and screen bacteria isolated from the xylem of eggplant, chilli, and* S. torvum* for their biocontrol activities against* R. solanacearum* and growth promotion abilities in eggplant.

## 2. Materials and Methods

### 2.1. Isolation of Xylem Residing Bacteria from Eggplant, Chilli, and* S. torvum*

#### 2.1.1. Collection of Xylem Sap

Apparently healthy plants were collected from the major vegetable growing locales in North Goa and South Goa districts of the coastal state of Goa, India. Eggplant samples were from two different varieties, namely, BW susceptible and BW resistant variety. Chilli samples were from the locally grown cultivar, which is moderately susceptible to BW. Wild eggplant* S. torvum* is known to be naturally resistant to BW and was sampled from a field in ICAR Research Complex for Goa, India. Stem pieces of 13–15 cm length were surface sterilized by dipping in 0.1% mercuric chloride for 1 min and rinsed several times in sterile water. Wash water used for rinsing each surface sterilized stem piece was plated onto Tryptic soy agar (TSA) (Hi Media Laboratories, Mumbai) to confirm surface sterilization of each stem piece. Xylem sap was extracted by vacuum infiltration as described earlier [7, 28]. Briefly, after the surface sterilization of stem, one cm piece from each end was discarded. Epidermis and cortex from each end were removed and the vascular cylinder was fitted to sterile glass tubing attached in a rubber cork. To the other end of the stem piece a sterile plastic tubing was attached that could hold at least 500 *μ*L of 1 X phosphate buffered saline (PBS) (NaCl 8 g/L, KCl 0.2 g/L, Na_2_HPO_4_·2H_2_O, 1.44 g/L, and KH_2_PO_4_ 0.24 g/L, pH 7.4). The cork with plant sample attached was then fitted onto a Buchner flask. For extraction of PBS through the xylem vessels a suction pressure 8 mbar was applied using a diaphragm pump MPC101Z (Ilmvac GmbH, Germany). A total of four successive infiltrations using 500 *μ*L of PBS were performed for each sample. The sap was collected directly in a sterile test tube placed inside the Buchner flask. Alternatively, maceration/trituration was performed for isolation of the XRB from young eggplant and chilli samples which had thin and soft stems. The epidermis and cortex from the surface sterilized stem piece were removed aseptically to expose the vascular bundles. The decorticated pieces were macerated in a sterile mortar and pestle using 2 mL of sterile 1X PBS.

#### 2.1.2. Isolation

One hundred *μ*L of the vacuum in-filtered sap or macerate was plated onto TSA or medium 523 [29]. The plates were incubated at 28 ± 2°C for 5 days. Different colonies from isolation plates were selected based on differences in their shape, color, and texture and purified onto medium 523. Pure cultures of the xylem residing bacteria (XRB) thus obtained were maintained at −80°C, as glycerol stocks for long term, and 4°C for temporary storage.

### 2.2. Amplified rDNA Restriction Analysis (ARDRA)

ARDRA was performed to determine the genetic diversity of the XRB in the collection. Genomic DNA from the XRB was extracted as described by Wilson [30]. Quality and quantity of the DNA were measured using Nanodrop 1000 (Thermo Scientific, USA). 16S rRNA gene was amplified using universal primers 27F (5′-AGAGTTTGATCCTGGCTCAG-3′) and 1492R (5′-GGTTACCTTGTTACGACTT-3′). Twenty *μ*L reaction mix contained 1X PCR buffer, 0.75 units of Taq DNA polymerase (Sigma Aldrich, USA), 200 *μ*M dNTPs (Sigma Aldrich, USA), 0.5*μ*M each primer (Chromous Biotech, Bangalore, India), and 50 ng/*μ*L of genomic DNA. Amplifications were carried out on Eppendorf Mastercycler Pro Thermal cycler (Eppendorf, Germany). Amplification cycle included a denaturation step of 94°C for 5 min followed by 32 cycles of denaturation at 94°C for 30*s*, annealing at 55°C for 40*s,* extension at 72°C for 1 min, and a final extension at 72°C for 10 min. The amplification of the 1500 bp PCR product was determined by electrophoresis on 0.8% agarose gel. Fifteen microliters of the PCR product was digested with one unit of* Msp*I (Thermo Scientific, USA) for 4 h at 37°C. Restriction fragments were separated on a 2% agarose gel in 1X Tris-acetate EDTA buffer containing 0.5 *μ*g/mL ethidium bromide at 60 volts for 2 h. Gel was documented using Alpha Imager (Alpha Innotech Inc., USA). ARDRA restriction fingerprints were compared visually and scored manually as 1 for presence and 0 for absence of fragment, and the binary data was entered in the NT Edit software version 1.1 b (Applied Biostatistics Inc. USA). The similarity matrix derived using the binary data of ARDRA restriction fragment was subjected to cluster analysis using unweighted pair group method for arithmetic average (UPGMA) using Dice coefficient in the NTSYSpc 2.02i software (Applied Biostatistics Inc. USA). Subsequent to analysis several clusters were obtained. Each cluster consisted of XRB having an identical restriction fragment profile. Strains having very unique restriction profile remained separable and independent clusters. Clusters obtained were denoted as haplotypes. Haplotypes were delineated at 80% similarity values of Dice coefficient and numbered as M80-1 to M80-38. Representative strains from each haplotype were identified by 16S rRNA gene sequencing.

### 2.3. Antagonism Towards* R. solanacearum*

#### 2.3.1. *In Vitro* Inhibition Bioassay

One hundred and sixty-seven XRB were screened for inhibition of the BW pathogen.* R. solanacearum* strain Rs-09-100 was isolated from BW infected eggplant cultivated in Goa, India, and was used for screening* in vitro* and* in planta*. Rs-09-100 belongs to phylotype I, race 1, and biovar 3 of the* R. solanacearum* species complex. The strain is pathogenic to eggplant cv.* Agassaim* and causes 100% wilt within 15 days after inoculation under greenhouse conditions (data not shown). Bioassay was performed by the agar well method as described by Ramesh and Phadke [27]. Briefly, single colony of* R. solanacearum* and XRB was grown in 5 mL CPG broth (Casein hydrolysate 1.0 g/L, Peptone, 10.0 g/L and Glucose, 5.0 g/L) and King's B broth (Peptone, 20.0 g/L, K_2_HPO_4_, 1.5 g/L, MgSO_4_· 7H_2_O, 1.5 g/L and Glycerol 10.0 mL/L), respectively, at 28 ± 2°C for 48 h with constant shaking at 140 rpm. One hundred and fifty microliters of* R. solanacearum* was seeded every 100 mL molten cooled King's B agar, mixed well, and poured into plates. After the plates solidified, three wells were made in each plate by removing a circular agar piece with the help of cork borer (8 mm diameter). Twenty-five *μ*L of culture broth of XRB containing 8.0 Log CFU/mL was added into each of the three wells. All the plates were incubated at 28 ± 2°C for 48 h. Plates were observed for inhibition of* R. solanacearum*. Zones of inhibition were measured as radius in mm from the edge of the agar well. Strains that were found antagonistic to* R. solanacearum* were screened for* in vitro* production of antagonistic compounds and plant growth promoting substances and identified by 16S rRNA gene sequencing.

### 2.4. Production of Volatile and Diffusible Antagonistic Substances by XRB

#### 2.4.1. Hydrogen Cyanide (HCN) Production

Antagonistic strains were tested for HCN production ability in presence of glycine as described by Saraf et al. [31], a slight modification being the use of broth for the HCN test. Immediately after inoculation of strains in King's B broth containing 4.4 g/L of glycine, sterile filter paper strips dipped in picric acid solution were introduced taking care that the strips did not touch the medium and walls of the tube. The tubes were sealed with parafilm and incubated at 28 ± 2°C for 4 days with constant shaking at 140 rpm. The color change of the filter paper strips from yellow to brick red during incubation indicated the production of HCN.

#### 2.4.2. Ammonia Production

To detect ammonia production, antagonistic XRB were grown in peptone water (peptone 20.0 g/L, NaCl 5.0 g/L) with constant shaking at 140 rpm for 48 h at 28 ± 2°C. Ammonia production was determined using Nessler's reagent as described by Marques et al. [32].

#### 2.4.3. Acetoin Production

Acetoin production by antagonistic isolates was tested in Voges Proskauer broth (peptone 7.0 g/L, K_2_HPO_4_ 5.0 g/L, dextrose 5.0 g/L pH 7.0). After incubation for 30 h at 28 ± 2°C at 140 rpm, one mL each of 5% *α* napthol and 40% KOH were added to the culture and mixed well. Appearance of red coloration indicated production of acetoin [33].

#### 2.4.4. Siderophore Production

Antagonistic XRB were tested for siderophore production on a medium containing chrome azurol S (CAS) [34]. Isolates producing orange haloes on the blue green colored medium after incubation at 28 ± 2°C for 48 h were positive for siderophore production.

### 2.5. Production of Growth Promoting Substances by the Antagonistic XRB

#### 2.5.1. Indole Acetic Acid (IAA) Production

Antagonistic strains were tested for their ability to produce phytohormone IAA in presence of tryptophan as described by Gordon and Paleg [35]. Briefly, strains were grown in nutrient broth amended with 100 mg/L of tryptophan for 30 h at 28 ± 2°C at 140 rpm. The supernatants were obtained by centrifugation at 6200 g for 10 min. One mL of supernatant was mixed with one mL of Salkowsky's reagent (50 mL 35% perchloric acid, 1 mL 0.5 M FeCl_3_). The mixture was allowed to stand at room temperature for five minutes and the absorbance was read at 530 nm. A standard curve was prepared using analytical grade IAA and the concentrations of IAA in the culture supernatants of XRB were estimated based on the curve.

#### 2.5.2. 1-Aminocyclopropane-1-carboxylate (ACC) Deaminase Activity

Antagonistic strains were tested for their ability to produce enzyme ACC deaminase as per the method described by Godinho et al. [36]. Strains were streaked on Dworkin and Foster's DF salts agar containing 3.0 mM ACC and incubated at 28 ± 2°C for 7 days. Ability of the strains to grow on the medium containing ACC as a sole nitrogen source was indicative of ACC deaminase production.

#### 2.5.3. Phosphate Solubilization

Antagonistic strains were tested for phosphate solubilization by a method described by Godinho et al. [36]. All strains were spot inoculated on Pikovskaya's agar plates (Hi Media Laboratories, Mumbai). Plates were incubated at 28 ± 2°C for 48 h. Transparent zones around the growth of XRB on the opaque white medium were indicative of solubilisation phosphate.

### 2.6. Greenhouse Experiments

#### 2.6.1. Biocontrol Efficacy (BCE) of Antagonistic XRB

Twenty-eight strains of XRB were selected based on* in vitro* inhibition of* R. solanacearum* in the agar well bioassay. Strains were evaluated for controlling BW in seedlings of wilt susceptible eggplant cv.* Agassaim* under greenhouse conditions. Thirty-day-old seedlings raised in nonsterile soil in greenhouse were transplanted in pots filled with standard nonsterile pot mixture (soil : sand : farmyard manure at 2 : 1 : 1 ratio). Ten mL suspension of antagonistic XRB (8.0 Log CFU/mL) in sterile 1 X PBS was applied per seedling by soil drenching. Each treatment consisted of two replicates with two pots per replication and five seedlings per pot. Twenty days after treatment with the antagonistic XRB the seedlings were challenged by inoculating 10 mL suspension of* R. solanacearum* strain Rs-09-100 (7.0 Log CFU/mL) by soil drenching. Plants not treated with XRB, but challenged with* R. solanacearum,* served as control. Plants were maintained with suitable watering and percentage of plants infected by wilt was noted until 25 days after challenging with* R. solanacearum*. Ability of the XRB to prevent wilt in eggplant was expressed as biocontrol efficacy (BCE) and was determined using the formula BCE = ([percent disease in control] − [percent disease in treatment]/percent disease in control) × 100 [37]. Strains with BCE greater than 25% were evaluated for their effect on growth in eggplant under greenhouse conditions.

#### 2.6.2. Growth Promotion Ability of XRB

Sixteen strains of XRB exhibiting biocontrol efficacies greater than 25% were studied for their effect on growth in eggplant. Ability to increase shoot length in wilt susceptible eggplant cv.* Agassaim* was used as a measure to evaluate their growth promotion efficacy under greenhouse conditions. Thirty-day-old seedlings raised in nonsterile soil in greenhouse were transplanted in pots filled with standard nonsterile pot mixture (soil : sand : farmyard manure at 2 : 1 : 1 ratio). Ten mL suspension of XRB (8.0 Log CFU/mL) in sterile 1 X PBS was applied per seedling by soil drenching. Each treatment consisted of two replicates with two pots per replication and five seedlings per pot. Plants were maintained with suitable watering and plant height was measured from the soil level to the shoot tip 40 days postinoculation. Ability of antagonistic XRB to increase shoot length in eggplant was expressed as growth promotion efficacy (GPE) using the formula ([shoot length increase in treatment] − [shoot length increase in control]/shoot length increase in control) × 100 [38].

### 2.7. 16S rRNA Gene Sequencing and Sequence Analysis

Representative strains from each of the 38 ARDRA haplotypes and XRB exhibiting antagonism to* R. solanacearum* were selected for identification. A total of 55 strains were chosen for identification. Fragments of the 16S rRNA gene of size 1500 bp were amplified as described above in the ARDRA section. Amplicons were purified using GeneJet PCR purification kit (Thermo Scientific, USA) and sequenced using 27F and 1492R primers (Xcelris Labs Pvt. Ltd., India). Partial 16S rRNA gene sequences (about 1200 nt) obtained were matched against the sequences available in the nucleotide database from National Center for Biotechnology Information (http://www.ncbi.nlm.nih.gov/BLAST) using the BLASTn (Basic Local Alignment Search Tool) program.

## 3. Results

### 3.1. Isolation of Bacteria from the Xylem of Eggplant, Chilli, and* S. torvum*

In this study, bacteria could be constantly isolated from the xylem of eggplant, chilli, and* S. torvum* by vacuum infiltration and maceration techniques. Bacterial counts from each isolation ranged from 10 to 10^2^ CFU/mL of xylem sap or macerate. Colonies appeared between 24 and 120 h of aerobic incubation at 28 ± 2°C. Amongst 167 isolates obtained, 99 were Gram-negative rods (59.28%) and 68 were Gram-positive bacteria (40.72%) comprising of 42 rods (61.76%), 25 cocci (36.76%), and one filamentous actinomycete (Table 1).

### 3.2. ARDRA Analysis

ARDRA generated three to six restriction fragments of the 16S rRNA gene amplified from the XRB. Analysis of ARDRA profiles by UPGMA using Dice's coefficient divided XRB with identical restriction profiles into several groups. At 80% similarity values of Dice coefficient, 167 strains of XRB were grouped into 38 haplotypes. Host based analysis of ARDRA revealed that 89 strains isolated from BW susceptible eggplant were grouped in 31 haplotypes, 36 strains from BW resistant eggplant into 17 haplotypes, 33 strains from chilli into 19 haplotypes, and 9 strains from* S. torvum* into 7 haplotypes, respectively (Table 1). A detailed representation of the ARDRA based analysis of 167 strains is presented in Table 2. Based on the ARDRA analysis of the collection of XRB, 153 strains were distributed over 24 different haplotypes and 14 XRB strains had unique profiles which formed 14 independent haplotypes with one strain in each. Antagonistic strains (*n* = 28) were distributed over 14 different ARDRA haplotypes wherein 6 antagonists formed independent haplotypes. Nonantagonistic strains were distributed over 24 haplotypes. Haplotypes M80-9 and M80-15 were shared amongst BW susceptible and resistant eggplant, chilli, and* S. torvum*. Eleven haplotypes were unique to BW susceptible eggplant. Haplotypes M80-1, M8-12, and M80-38 were unique to BW resistant eggplant whereas haplotypes M80-13, M80-19, and M80-26 comprised of strains isolated from chilli. Haplotype M80-36 had a strain isolated from* S. torvum*. Other haplotypes were a combination of strains isolated from different plant species. These results indicate that bacterial communities from the xylem of mainly eggplant and chilli cultivated in different locations in Goa comprise of diverse bacteria. However it is observed that each ARDRA group consists of bacteria from different plant species and plants collected from different locales. In addition there are certain XRB unshared between each of the plant species that form unique haplotypes. Moreover, strains with biocontrol ability (BCE > 25%) were restricted to only 8 haplotypes, namely, M80-6, M80-7, M80-10, M80-15, M80-20, M80-29, M80-31, and M80-36 (Table 2). Haplotypes M80-6, M80-7, M80-29, M80-31, M80-36, and M80-38 comprised of XRB with GPE > 10%. Interestingly, the strains with BCE > 25% and GPE > 10% within these haplotypes were from BW resistant eggplant (Table 1).

### 3.3. Antagonism towards* R. solanacearum*

#### 3.3.1. *In Vitro* Bioassay and Production of Volatile and Diffusible Antagonistic Compounds

Plate based bioassay was used for rapid screening of antagonism of XRB towards* R. solanacearum* strain Rs-09-100. Results of the* in vitro* screening against* R. solanacearum* revealed that 28 amongst 167 XRB exhibited antagonism towards the pathogen (Table 3). Amongst the antagonists, 16 were strains from BW susceptible eggplant, 6 from BW resistant eggplant, and 3 each from chilli and* S. torvum* (Table 1). Amongst the 28 antagonists, 7 strains, namely, XB62, XB99, XB100, XB114, XB122, XB196, XB197, and XB202 formed larger inhibition zones against* R. solanacearum* ranging from 4.0 mm to 8.17 mm (Table 3). The majority of the antagonistic strains (*n* = 16) produced inhibition zones ranging from 2.0 mm to 3.83 mm. XB27, XB134, XB165, and XB169 formed smaller inhibition zones ranging from 1.5 mm to 2.0 mm. However, 139 strains of XRB did not inhibit* R. solanacearum* in the bioassay test. Twenty-eight antagonistic XRB were screened for production of volatile inhibitory compounds, namely, acetoin, HCN, ammonia, and diffusible siderophore molecules* in vitro* (Table 3). Acetoin production was observed in 32.14% of the isolates. Bacterial isolates XB7 and XB122 were found to produce both HCN as well as siderophores. XB62, XB93, and XB170 produced HCN whereas XB114, XB140, and XB203 produced siderophores only. XB93, XB99, XB123, XB134, and XB140 produced ammonia.

### 3.4. Production of Plant Growth Promoting Substances by Antagonistic XRB

Results of the screening of antagonistic XRB for* in vitro* production of several plant growth promoting compounds is presented in Table 3. Majority of the antagonistic strains produced the phytohormone IAA with concentrations ranging from 15.91 *μ*g/mL to 645.91 *μ*g/mL. XB202 was found to be the best ACC deaminase producing strain based on its luxuriant growth on DF salts medium supplemented with 3.0 mM ACC as sole nitrogen source. Other ACC deaminase producing strains include XB1, XB62, XB86, and XB140. Scarce growth of XB165 and XB200 was observed on DF salts medium. 64.28% of the strains produced phosphate solubilizing organic acids as indicated by clear haloes on Pikovskaya's agar plate.

### 3.5. Greenhouse Experiments

#### 3.5.1. Suppression of Bacterial Wilt by Antagonistic XRB

**Figure 1:**
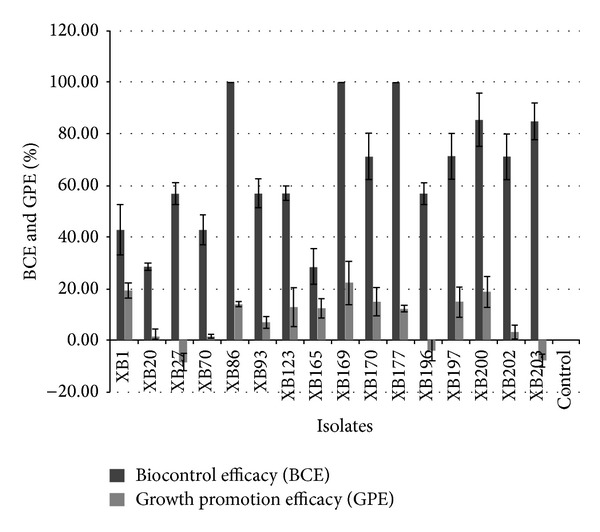
Biocontrol and plant growth promotion efficacies of select XRB in eggplant. BCE: biocontrol efficacy determined 25 days after challenging with* R. solanacearum*; GPE: growth promotion efficacy determined 40 days after treatment with XRB. Percent BCE and GPE calculated using formulae BCE = ([percent disease in control] − [percent disease in treatment]/percent disease in control) × 100 and GPE = ([shoot length increase in treatment] − [shoot length increase in control]/shoot length increase in control) × 100, respectively. Uninoculated control had BCE and GPE values of 0.00; XB86, XB169, and XB177 had BCE of 100.00% in all the replications. Bars indicate mean values of % BCE and GPE; error bars indicate standard deviation.

Ability to suppress BW was assessed as the difference in the percentage of wilt in XRB treated plants with respect to wilt in untreated control and was expressed as the biocontrol efficacy (BCE) of the antagonists. BCE of 16 strains with values ranging from 28.6 to 100% is presented in Figure 1. Plants treated with strains XB86, XB169, and XB177 were free from BW and hence recorded 100% biocontrol efficacy. Treatments with XB170, XB197, XB200, XB202, and XB203 recorded 30 percent or less wilt incidence (70% to 85% BCE). XB1, XB27, XB70, XB93, and XB123 treatments recorded BCE between 42.9 and 57.1%. BCE of 28.6% was recorded in XB20 and XB165 treatments. However, 12 antagonistic XRB recorded BCE of 25% and were least effective in wilt protection. Further it is observed that all the antagonistic strains originating from BW resistant eggplant and* S. torvum* exhibited BCE greater than 25%. Five antagonistic strains from BW susceptible eggplant and three from chilli were effective in preventing wilt in eggplant (Table 1).

#### 3.5.2. Growth Promotion by Antagonistic XRB

Increase in shoot length of eggplant (40 days after treatment) observed in XRB treated plants in relation to untreated control was expressed as growth promotion efficacy (GPE) and is shown in Figure 1. Amongst the 16 strains which were effective in preventing wilt, six strains exhibited the highest increase in shoot length as indicated by their GPE values in the range of 13.9–22.3%. Seven XRB recorded a GPE value ranging from 1.4 to 12.9%. However, strains XB27, XB196, and XB203 stunted shoot growth in eggplant, in comparison to untreated control. When the source of XRB is considered, 55.55% strains that exhibited GPE greater than 10% were isolated from BW resistant eggplant. Whereas, only two antagonists from BW susceptible plant and one each from chilli and* S. torvum* were able to promote growth in eggplant (Table 1). Strains with GPE > 10% belonged to six different ARDRA haplotypes (Table 2).

### 3.6. Identification of XRB by 16S rRNA Gene Sequencing

**Figure 2:**
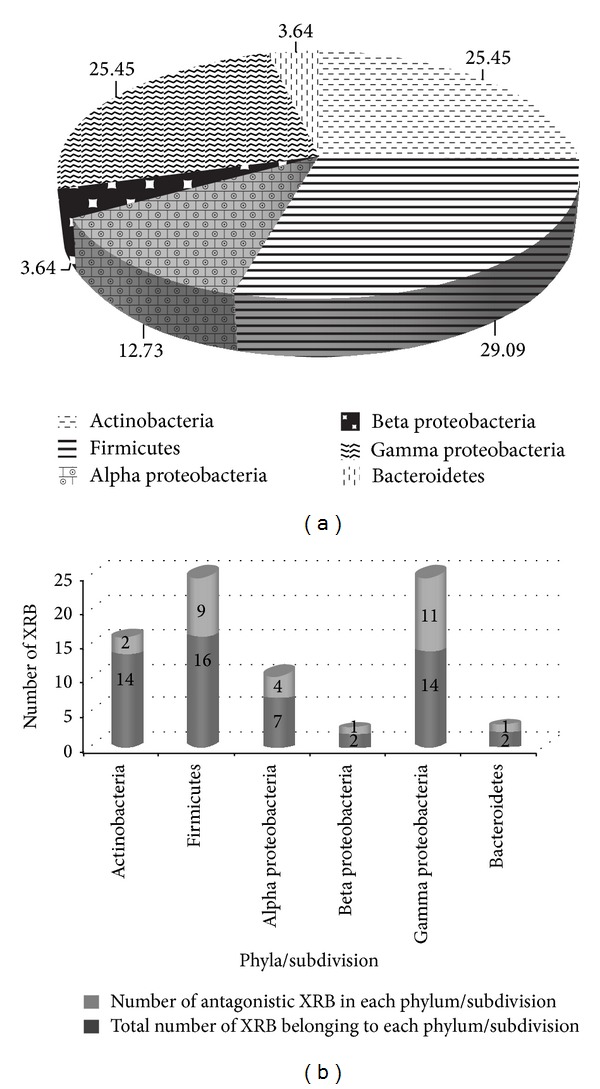
(a) Distribution of XRB (phylum/subdivision level) identified by 16S rRNA gene sequencing. Values indicate percentages of strains belonging to each phyla/subdivision amongst the 55 identified strains. (b) Distribution of antagonistic and nonantagonistic strains of XRB in each phylum/subdivision.

16S rRNA gene sequences of XRB were used to identify the diverse xylem inhabitants and antagonistic strains. Identity of 55 XRB based on 16S rRNA gene sequencing and their GenBank accessions are presented in Table 4. Overall, 23 different genera of bacteria were identified. Major genera identified are* Bacillus* sp. (11 strains),* Enterobacter* sp. (6 strains),* Microbacterium* sp. (5 strains),* Staphylococcus* sp. (5 strains),* Pseudomonas* sp. (5 strains), and* Agrobacterium* sp. (3 strains). Additional genera identified including* Micrococcus* sp.,* Sphingomonas* sp.,* Flavobacterium* sp.,* Chryseobacterium* sp.,* Burkholderia* sp., and* Xenophilus* sp. are listed in Table 4. Based on the identification, phylum Proteobacteria consisting of Gram-negative bacteria of subdivisions* Alpha* Proteobacteria (12.73%),* Beta* Proteobacteria (3.64%), and* Gamma* Proteobacteria (25.45%) were predominant (41.81% strains identified), followed by phyla Firmicutes (29.09%), Actinobacteria (25.45%), and Bacteroidetes (3.64%) (Figure 2(a)).

Eleven antagonistic strains identified belonged to* Gamma* subdivision of Proteobacteria consisting of five strainseach of* Enterobacter* sp. and fluorescent and nonfluorescent* Pseudomonas *sp. and one strain of* Pantoea eucrina *(XB126) (Figure 2(b)). Nine antagonists identified were of phyla Firmicutes consisting of* Staphylococcus* sp. (5 strains) and* Bacillus* sp. (4 strains).* Agrobacterium* strains XB1, XB86, and XB165,* Sphingomonas* sp. (XB197) of the* Alpha *Proteobacteria,* Streptomyces* sp. (XB200), and* Janibacter melonis* (XB70) of phyla Actinobacteria were found to be antagonistic. Additional antagonistic XRB include* Burkholderia* sp. (XB140) of**β** Proteobacteria and* Flavobacterium* sp. (XB203) of phyla Bacteroidetes (Figure 2(b)).

### 3.7. Statistical Analysis

The statistical analysis of percentage wilts and shoot length of eggplant was performed using Web Agri Statistical Package (WASP) version 2.0 (http://www.icargoa.res.in/wasp2.0/index/php).

## 4. Discussion

Eggplant and chilli not only are of economic and cultural importance but also are common ingredients in the cuisine throughout India. In the coastal state of Goa,* R. solanacearum* has been reported to be a destructive pathogen in cultivation of eggplant and chilli [4]. Isolation of biocontrol agents against the BW pathogen has been commonly restricted to endophytic tissue and plant rhizosphere [26, 27]. Studies on xylem colonizing endophytes were undertaken because we speculated existence of interactions between the XRB and vascular wilt pathogen* R. solanacearum* during xylem colonization. Our study reveals the diversity, biocontrol potential, and identity of endophytic xylem colonizers from solanaceous crops cultivated in Goa, India. A total of 167 bacteria were isolated from the xylem of eggplant, chilli, and* S. torvum* with Gram-negative bacteria (59.28%) predominating in the collection. Congruent to our observation, Gardner et al. [7] and Bell et al. [11] have earlier reported isolation of more number of Gram-negative rod shaped bacteria from xylem of citrus and grapevine using vacuum infiltration. Scholander pressure bomb was found to be useful in extraction of diverse bacterial genera from xylem tissues in contrast to trituration methods that yielded higher number of Gram-positive rod shaped bacteria [12]. Though, Scholander pressure bomb was not used in this study, a combination of vacuum infiltration [7, 11] and trituration of decorticated stems [10, 12] was employed with an aim to isolate diverse XRB from xylem tissues. This is the first study reporting the use of vacuum infiltration and trituration methods for isolating xylem residing bacteria from eggplant, chilli, and* S. torvum*.

Traditionally bacteria have been characterized and grouped based on colony morphology and biochemical tests. However, whole genome fingerprinting or PCR-RFLP based methods are rapid tools for determining genetic diversity of bacteria in a given collection. ARDRA which is a type of PCR based RFLP method has been used widely in estimating genetic diversity endophytic bacterial populations and clustering genetically similar strains [39–41]. In addition to earlier reports, our study demonstrates the usefulness of ARDRA as a tool to cluster genetically identical strains of endophytic XRB isolated from eggplant, chilli, and* S. torvum*. In our study 91.61% strains (*n* = 153) were grouped in 24 haplotypes by using ARDRA. The majority of these haplotypes represent a combination of XRB isolated from different solanaceous plants from diverse locales. These results indicate that xylem of eggplant, chilli, and* S. torvum* largely bears similar population of XRB which can efficiently cross colonize eggplant, chilli, or* S. torvum*. Nevertheless, 8.39% strains had unique ARDRA fingerprint and formed separable haplotypes. This observation leads to a conclusion that a minor population of xylem inhabitants are restricted to a specific plant species and cannot easily cross colonize xylem of other solanaceous plants. Plants are known to selectively support endophytic colonization by specific bacteria [14]. However, factors that determine the selection of xylem colonists or the ability of XRB to colonize eggplant, chilli, and* S. torvum* in this study remain unknown. Interestingly, the structure of endophytic community in* Nicotiana attenuata* a member of Solanaceae family is shown to be influenced by soil composition and ethylene homeostasis [40]. Earlier evidence has shown that colonization by endophytic bacteria is also governed by plant genotype as well as root exudates [42].

Only 16.77% XRB out of 167 were antagonistic to* R. solanacearum* based on* in vitro* assays. Antagonistic XRB produced volatile and diffusible inhibitory compounds, namely, HCN, ammonia, and acetoin and siderophores. These substances have been long known to be involved in disease suppression and indirect growth promotion in plants [43–46]. These mechanisms possibly played a role in the evident biocontrol effect against BW exhibited by the XRB in the greenhouse screening. Endophytic bacteria have been known to have plant growth promoting traits, namely, production of IAA, ACC deaminase, and phosphate solubilization [25, 33, 36]. These traits were detected in the majority of antagonistic XRB tested in this study and may have resulted in the observed increase in shoot length of eggplant in our greenhouse experiments. In contrast, strains XB20, XB196, and XB203 suppressed growth in eggplant under greenhouse conditions; however no visible symptoms of disease were observed. Vascular plugging and production of certain metabolites toxic to plant cells, but not cell viability, may have resulted in stunted shoot in eggplant [11, 47].

Evaluation of efficacy of antagonistic organisms to suppress the plant diseases under greenhouse conditions is one of the key steps for selecting a potential biocontrol agent for disease management [48]. Endophytic strains from BW susceptible varieties of eggplant have been shown to prevent wilt and promote growth in eggplant earlier [27]. Our greenhouse screening shows that 38.46% of antagonistic XRB with biocontrol efficacies greater than 40% were isolates from BW resistant varieties of eggplant. This raises a question whether the bacteria from resistant varieties are involved in BW resistance and whether BW resistant varieties are able to selectively influence xylem colonization by antagonistic bacteria? Presence of higher number of endophytes with antagonistic abilities was reported in BW resistant varieties of tomato as compared to susceptible varieties, and their role in resistance to BW was proposed [49]. Similar observations on correlation of resistance of potato to soft rot and endophytic bacteria have been reported [50]. Thus BW resistant varieties can be considered a better host for isolating potential biocontrol strains for management of bacterial wilt.

Identification of 55 XRB strains by 16S rRNA gene sequencing revealed the presence of 23 diverse genera of bacteria belonging to 4 phyla of Eubacteria. Strains belonging to phyla Firmicutes, Actinobacteria, and *γ* subdivision of Proteobacteria were the major xylem colonists identified in this study. Several genera of bacteria belonging to these phyla have also been reported to be present in endophytic tissues and xylem of a variety of other agricultural and horticultural plant species [14, 51]. In addition, the majority of the antagonists identified belonged to* Enterobacter* sp.,* Pseudomonas* sp.,* Bacillus* sp., and* Staphylococcus* sp. Congruent to our results, several researchers have reported bacteria isolated from solanaceous crops and belonging to similar genera to be antagonistic to* R. solanacearum* [26, 51–53]. However,* Flavobacterium* sp.and* Janibacter melonis* identified in this study have never been previously reported to be inhibitory to* R. solanacearum*. Large population of the xylem inhabiting bacterial flora accounting for 83.23% exhibited no antagonism towards* R. solanacearum*. Nonantagonistic XRB were identified predominantly as* Microbacterium* sp. Endophytic persistence and nematicidal activities of* Microbacterium* sp. have been reported [54, 55]. Therefore the collection of XRB isolated in this study can be screened for inhibitory activities against other important agricultural pests.

Though few strains, namely, XB86, XB169, and XB177 exhibited plant beneficial properties in this study their usefulness in plant disease control remains to be seen. XB86 has been identified as* Agrobacterium tumefaciens,* the crown gall disease pathogen, and its deployment as biocontrol agent is uncertain. XB169 (*Staphylococcus gallinarum*) and XB177 (*Bacillus cereus*) are reported as opportunistic animal pathogens and thus unsuitable for field applications.* Streptomyces* sp. has earlier been reported as antagonistic to* R. solanacearum* and tested for management of wilt in potato and tomato [56, 57]. XB200 (*Streptomyces* sp.) is one of the XRB high BCE and GPE; it could be explored further for biocontrol of bacterial wilt after additional characterization and field evaluation.

## 5. Conclusion

This study is the first report on the identity of novel and diverse XRB colonizing the xylem of eggplant, chilli, and* S. torvum*. XRB particularly from BW resistant varieties were found to protect eggplant from bacterial wilt and enhanced growth in eggplant in the greenhouse screening. Therefore the repertoire of XRB reported in this study may be useful for cultivation of eggplant in BW affected areas.

## Figures and Tables

**Table 1:** Overview of diversity and functionality of XRB obtained from eggplant, chilli, and *S. torvum*.

Plant host	Number of samples	Total isolates obtained	Gram-negative	Gram-positive	Haplotypes^a^ obtained	Antagonistic strains^b^	Antagonistic strains with BCE > 25%	Biocontrol strains with GPE > 10%	Method of isolation
BW susceptible eggplant	24	89	53	36	31	16	5	2	VI or M
BW resistant eggplant	12	36	20	16	17	6	6	5	VI or M
Chilli (*Capsicum annuum*)	7	33	20	13	19	3	2	1	M only
*Solanum torvum *	2	9	6	3	7	3	3	1	VI only

Total	45	167	99	68		28	16	9	

^a^Haplotypes were determined using ARDRA.

^b^Antagonism to *R. solanacearum* tested *in vitro* as described in Section 2.

BCE (biocontrol efficacy) and GPE (growth promotion efficacy) were determined under greenhouse conditions in eggplant as described in Section 2.

VI: Vacuum infiltration, M: maceration.

**Table 2:** Haplotypes of XRB based on ARDRA with *Msp*I at 80% similarity, plant host, biocontrol, and growth promotion activities.

Haplotype number^a^	Number of strains in each haplotype	Plant host	Strains selected for identification	Number of biocontrol strains with BCE > 25%	Number of strains with GPE > 10%
M80-1	1	RE	XB159	0	0
M80-2	1	SE	XB34	0	0
M80-3	1	SE	XB66	0	0
M80-4	2	SE, C	XB40	0	0
M80-5*	1	SE	XB140	0	0
M80-6*	10	SE, RE, C	XB177, XB157, XB93, XB169, XB153, XB170	4	3
M80-7*	6	SE, RE, C	XB1, XB86	2	2
M80-8	4	SE, RE	XB22, XB190	0	0
M80-9*	8	SE, RE, ST, C	XB99, XB100	0	0
M80-10*	10	SE, RE, C	XB196, XB103	1	0
M80-11	9	SE, RE, C	XB137	0	0
M80-12	1	RE	XB158	0	0
M80-13	1	C	XB87	0	0
M80-14	5	SE, C	XB88	0	0
M80-15*	5	SE, RE, ST, C	XB70	1	0
M80-16	3	SE	XB47	0	0
M80-17	2	SE	XB35	0	0
M80-18	1	SE	XB41	0	0
M80-19	2	C	XB94	0	0
M80-20*	13	SE, RE, C	XB8, XB20, XB27	2	0
M80-21	5	SE, ST, C	XB25	0	0
M80-22	1	SE	XB53	0	0
M80-23	5	SE, RE, C	XB98	0	0
M80-24	11	SE, RE, ST	XB161	0	0
M80-25	4	SE, RE	XB188	0	0
M80-26	1	C	XB168	0	0
M80-27*	2	SE, C	XB126, XB7	0	0
M80-28*	5	SE, RE	XB122	0	0
M80-29*	9	SE, RE, C	XB165	1	1
M80-30	15	SE, RE, C	XB167, XB109	0	0
M80-31*	11	SE, ST, C	XB123, XB203, XB62, XB114, XB202	3	1
M80-32	5	SE, ST, C	XB92	0	0
M80-33	1	SE	XB37	0	0
M80-34	1	SE	XB36	0	0
M80-35	2	SE	XB64	0	0
M80-36*	1	ST	XB200	1	1
M80-37*	1	SE	XB134	0	0
M80-38*	1	RE	XB197	1	1

^a^Haplotype based on ARDRA analysis using *Msp*I at 80% similarity values of Dice coefficient.

*Haplotype comprises of bacteria showing antagonistic property in the bioassays.

SE: bacterial wilt susceptible eggplant, RE: bacterial wilt resistant eggplant, ST: *Solanum torvum*, C: chilli plant.

BCE: biocontrol efficacy, GPE: growth promotion efficacy determined as described in Section 2.

**Table 3:** * In vitro* inhibition of *R. solanacearum* and production of antagonistic compounds and plant growth promoting substances by XRB.

Strain	Haplotype	Inhibition of *R. solanacearum *	Volatile and diffusible inhibitory compounds				Plant growth promoting substances		
		Radius in mm	HCN	Ammonia	Acetoin	Siderophore	Phosphate solubilisation	ACC deaminase^a^	IAA^b^ *μ*g/mL
XB1	M80-7	2.83 ± 0.29	−	−	−	−	−	+++	105.00 ± 7.07
XB7	M80-27	3.33 ± 0.58	+	−	−	+	+	−	47.73 ± 3.21
XB8	M80-20	3.33 ± 0.29	−	−	−	−	+	−	19.09 ± 1.29
XB20	M80-20	3.27 ± 0.40	−	−	−	−	+	−	25.45 ± 1.71
XB27	M80-20	1.83 ± 0.29	−	−	−	−	+	−	15.91 ± 1.07
XB62	M80-31	8.17 ± 0.76	+	−	−	−	+	++++	73.18 ± 4.93
XB70	M80-15	3.83 ± 0.58	−	−	−	−	−	−	171.82 ± 11.57
XB86	M80-7	2.33 ± 0.76	−	−	−	−	−	+++	66.82 ± 4.50
XB93	M80-6	3.17 ± 0.29	+	−	+	−	+	−	57.27 ± 3.86
XB99	M80-9	6.83 ± 0.58	−	+	+	−	+	−	321.36 ± 21.64
XB100	M80-9	4.50 ± 0.50	−	+	+	−	+	−	238.64 ± 16.07
XB114	M80-31	6.50 ± 0.50	−	−	−	+	+	−	89.09 ± 6.00
XB122	M80-28	4.33 ± 0.58	+	−	−	+	+	−	66.82 ± 4.50
XB123	M80-31	3.00 ± 0.87	−	+	+	−	+	−	416.82 ± 28.07
XB126	M80-27	2.83 ± 0.29	−	−	+	−	+	−	60.45 ± 4.07
XB134	M80-37	1.57 ± 0.12	−	+	+	−	+	−	645.91 ± 43.50
XB140	M80-5	3.67 ± 0.76	−	+	−	+	+	++++	50.91 ± 3.43
XB153	M80-6	3.17 ± 0.29	−	−	+	−	+	−	155.91 ± 10.50
XB157	M80-6	2.50 ± 1.00	−	−	+	−	+	−	184.55 ± 12.43
XB165	M80-29	1.57 ± 0.12	−	−	−	−	−	+	190.91 ± 12.86
XB169	M80-6	1.53 ± 0.06	−	−	−	−	−	−	15.91 ± 1.07
XB170	M80-6	3.51 ± 0.02	+	−	−	−	+	−	28.64 ± 1.93
XB177	M80-6	1.99 ± 0.49	−	−	−	−	−	−	76.36 ± 5.14
XB196	M80-10	3.93 ± 0.12	−	−	−	−	−	−	35.00 ± 2.36
XB197	M80-38	4.33 ± 0.29	−	−	+	−	−	−	41.36 ± 2.79
XB200	M80-36	1.87 ± 0.23	−	−	−	−	−	++	168.64 ± 11.36
XB202	M80-31	4.67 ± 0.21	−	−	−	−	+	+++	82.73 ± 5.57
XB203	M80-31	3.67 ± 0.29	−	−	−	−	−	−	70.00 ± 4.71

Inhibition zones are mean of three replications and showing standard deviation. Inhibition zone was measured as radius from the outer edge of well. All the experiments were conducted at 28 ± 2°C. + indicates presence of trait; − indicates absence of trait in plate based assays. ^a^Levels of ACC deaminase activities based on growth on plate based assay denoted as + for less growth, +++ for moderate growth, +++ for luxuriant growth, and ++++ for highly luxuriant growth; ^b^IAA was estimated in culture filtrate and expressed as *μ*g/mL using analytical grade IAA as standard; values indicate mean and standard deviation.

**Table 4:** List of XRB identified by partial 16S rRNA gene sequencing their plant host, accession numbers, closest NCBI match, and % similarity.

Strain	Plant host	Accession number	Closest NCBI match	% similarity
XB1*	SE	KF447383	*Agrobacterium tumefaciens *	99
XB7*	SE	KF447384	*Pseudomonas aeruginosa *	99
XB8*	SE	KF447385	*Staphylococcus haemolyticus *	99
XB20*	SE	KF447386	*Staphylococcus haemolyticus *	100
XB22	SE	KF447387	*Brevibacterium casei *	99
XB25	C	KF447388	*Enterobacter* sp.	95
XB27*	SE	KF447389	*Staphylococcus haemolyticus *	99
XB34	SE	KF447390	*Curtobacterium* sp.	99
XB35	SE	KF447391	*Microbacterium* sp.	99
XB36	SE	KF447392	*Xenophilus* sp.	99
XB37	SE	KF447393	*Chryseobacterium* sp.	99
XB40	SE	KF447394	*Micrococcus luteus *	89
XB41	SE	KF447395	*Bacillus* sp.	99
XB47	SE	KF447396	*Bacillus* sp.	99
XB53	SE	KF447397	*Micrococcus* sp.	99
XB62*	SE	KF447398	*Pseudomonas* sp.	99
XB64	SE	KF447399	*Bacillus thuringiensis *	100
XB66	SE	KF447400	*Pectobacterium carotovorum *	99
XB70*	SE	KF447401	*Janibacter melonis *	99
XB86*	C	KF447402	*Agrobacterium tumefaciens *	99
XB87	C	KF447403	*Bacillus barbaricus *	99
XB88	C	KF447404	*Bacillus* sp.	99
XB92	C	KF447405	*Brevundimonas vesicularis *	99
XB93*	C	KF447406	*Bacillus safensis *	100
XB94	C	KF447407	*Bacillus* sp.	100
XB98	C	KF447408	*Microbacterium* sp.	100
XB99*	SE	KF447409	*Enterobacter* sp.	99
XB100*	SE	KF447410	*Enterobacter cloacae *	98
XB103	SE	KF447411	*Brachybacterium phenoliresistens *	99
XB109	SE	KF447412	*Rhodococcus corynebacterioides *	99
XB114*	SE	KF447413	*Pseudomonas stutzeri *	99
XB122*	SE	KF913446	*Pseudomonas aeruginosa *	100
XB123*	SE	KF447414	*Enterobacter* sp.	99
XB126*	C	KF447415	*Pantoea eucrina *	99
XB134*	SE	KF447416	*Enterobacter* sp.	99
XB137	SE	KF447417	*Klebsiella* sp.	99
XB140*	SE	KF447418	*Burkholderia* sp.	99
XB153*	SE	KF447419	*Bacillus amyloliquefaciens *	99
XB157*	SE	KF447420	*Bacillus subtilis *	100
XB158	RE	KF447421	*Bosea* sp.	99
XB159	RE	KF447422	*Microbacterium xylanilyticum *	99
XB161	RE	KF447423	*Microbacterium aurum *	99
XB165*	RE	KF447424	*Agrobacterium* sp.	99
XB167	RE	KF447425	*Microbacterium aurum *	99
XB168	RE	KF447426	*Bacillus aryabhattai *	100
XB169*	RE	KF447427	*Staphylococcus gallinarum *	99
XB170*	RE	KF447428	*Staphylococcus* sp.	99
XB177*	RE	KF447429	*Bacillus cereus *	99
XB188	RE	KF447430	*Sphingomonas* sp.	99
XB190	RE	KF447431	*Brevibacterium casei *	99
XB196*	RE	KF447432	*Enterobacter kobei *	99
XB197*	RE	KF447433	*Sphingomonas* sp.	98
XB200*	ST	KF913447	*Streptomyces* sp.	100
XB202*	ST	KF447434	*Pseudomonas* sp.	99
XB203*	ST	KF447435	*Flavobacterium* sp.	99

*Strain is antagonistic to *R. solanacearum* based on bioassay.

SE: bacterial wilt susceptible eggplant, RE: bacterial wilt resistant eggplant, C: chilli, ST: *Solanum torvum*.
